# Distinct patterns of natural selection determine sub-population structure in the fire blight pathogen, *Erwinia amylovora*

**DOI:** 10.1038/s41598-019-50589-z

**Published:** 2019-09-30

**Authors:** Jugpreet Singh, Awais Khan

**Affiliations:** Plant Pathology and Plant-Microbe Biology Section, Cornell University, Geneva, NY 14456 USA

**Keywords:** Phylogenomics, Microbial genetics

## Abstract

The fire blight pathogen*, Erwinia amylovora* (EA), causes significant economic losses in rosaceae fruit crops. Recent genome sequencing efforts have explored genetic variation, population structure, and virulence levels in EA strains. However, the genomic aspects of population bottlenecks and selection pressure from geographical isolation, host range, and management practices are yet unexplored. We conducted a comprehensive analysis of whole genome sequences of 41 strains to study genetic diversity, population structure, and the nature of selection affecting sub-population differentiation in EA. We detected 72,741 SNPs and 2,500 Indels, representing about six-fold more diversity than previous reports. Moreover, nonsynonymous substitutions were identified across the effector regions, suggesting a role in defining virulence of specific strains. EA plasmids had more diversity than the chromosome sequence. Population structure analysis identified three distinct sub-groups in EA strains, with North American strains displaying highest genetic diversity. A five kilobase genomic window scan showed differences in genomic diversity and selection pressure between these three sub-groups. This analysis also highlighted the role of purifying and balancing selection in shaping EA genome structure. Our analysis provides novel insights into the genomic diversity and selection forces accompanying EA population differentiation.

## Introduction

*Erwinia amylovora* (EA), a gram-negative bacterium, was the first bacterial pathogen shown to cause disease in plants^1–3^. After first being reported in 1780 in New York, it spread across other apple and pear producing regions of the world, including New Zealand, United Kingdom, Europe, and the Middle East^4^. EA causes fire blight, which is a severe threat to apple and pear production worldwide, leading to significant economic losses^4–6^. After first occurrence, fire blight remains a highly prevalent and infectious disease in apple, pear, and other rosaceae host plants, and can kill an entire orchard within one growing season^4^. In comparison, some EA strains infecting *Rubus* species appear to be less prevalent^7,8^. The bacteria enter into plants through natural openings or wounded plant parts including rootstocks, shoots, leaves, flowers, and fruits to cause initial infection, from where they can spread through xylem vessels to infect and kill the entire plant^9–11^. Development of necrotic lesions on various plant parts, bacterial ooze, wood cankers, and molding of shoot curvature (shepherd’s crook) are typical symptoms of fire blight. Use of disease forecasting models, chemicals and pruning of infected twigs present some preventive measures against fire blight infection. However, knowing the genome-wide polymorphism in diverse bacterial strains provides better understanding of EA virulence, evolution, and spread for devising appropriate disease management solutions.

Genome sequencing of different strains has shown that EA has a small genome size of approximately 3.8 megabases^12,13^. The coding sequence represents about 86% of the entire genome, and includes conserved hypothetical proteins, mobile elements, pseudogenes, and genes involved in cellular envelope biosynthesis/modification and signal transduction^12^. Unlike other phytopathogenic bacteria, the EA genome lacks enzymes related to cell wall degradation and low molecular weight toxins^2^. The EA genome carries three Hrp T3SS (Hypersensitive reaction and pathogenicity, Type III secretion systems) gene clusters and three eop2, HopPtoC, and AvrRpt2 single gene effectors^13^. Presence of Hrp T3SS effectors enable the bacteria to deliver virulent molecules into the cytosol of host plants, which interact with DspA/E proteins for pathogenicity and hypersensitive response in resistant plants^9,10,14–16^. These interactions result in exopolysaccharide synthesis to form biofilm for bacterial colonization, movement and pathogenicity in host plants^11,16,17^. Likewise, an induced deletion and single nucleotide change in the AvrRpt2 effector reduces the EA infection on pear fruits^18–20^, although the role of the remaining two singleton effectors on EA virulence is not clear.

The EA genome also contains three clustered regularly interspaced short palindromic repeat (CRISPR) regions^13^ for immunity against bacteriophages. The distribution of spacers in the CRISPR loci have been frequently used to classify diverse EA strains^21,22^. For example, an analysis of CRISPR regions identified three distinct spacer patterns in EA that were able to distinguish apple and pear infecting strains from eastern and western U.S^21^. In addition, the *Rubus*-infecting (RI) strains showed distinct CRISPR patterns against apple and pear infecting strains^21,22^. Similar analysis of tandem repeats also differentiated three distinct groups in a worldwide collection of 833 EA strains^23^. However, a restricted genome analysis provides only limited information about genetic diversity and precise phylogenetic structure in EA strains. Recently, high coverage resequencing and comparison of 12 EA strains revealed about 89% conserved core genes with slight amino acid variation^24^. Analysis of a larger set of strains from diverse geographical origins reported about 30-fold more genetic diversity^25^, suggesting the presence of additional genetic variation in the Erwinia populations. The phylogenetic analysis not only classified the Spiraeoideae-infecting (SI) strains from RI strains^24^, but also underlined the effect of geographical distinction between widely prevalent and more local strains^25^. The genetic diversity in SI strains was comparatively less than in RI strains. In addition, North American EA strains appear to be more diverse than European strains^25^. Although these studies have provided some information about genetic diversity and phylogeny of EA, collection and analysis of additional strains can discover novel variants^25^ and improve the genetic variation map of this pathogen.

In addition to chromosomal DNA, plant pathogenic bacteria possess plasmids of different sizes that enhance their fitness, adaptability and genetic evolution as well as contribute towards virulence and development of resistance to certain antibiotics, and are therefore critical targets for genome analysis^26,27^. EA has also been reported to acquire new genes through horizontal gene transfer. This process of genetic exchange enables rapid evolution of the genome of EA and increases its genetic plasticity, leading to advantages in host–pathogen interactions during fire blight infection^26^. The diversity in host range, aggressiveness, virulence levels, and fitness of EA may primarily be attributed to the genome content of plasmids^13,28–30^. Several plasmids have been identified in different EA strains from different geographical areas^12,27,31–33^. The non-conjugative ‘pEA29’ plasmid is commonly present in all EA strains, but some strains lack ‘pEA29’^33–35^ or carry additional plasmids^25,27^. For example, another plasmid ‘pEA34’ was identified in strains from Michigan that harbors two streptomycin-resistant genes^32^ highlighting the role of plasmid associated variation for overcoming local selection pressure. Streptomycin is one of the most effective antibiotics used to reduce the incidence of blossom blight in the U.S. EA strains have developed two distinct chromosome and plasmid level genetic mechanisms to confer streptomycin resistance, (1) Point mutations in codon 43 of rpsL gene encoding ribosomal protein S12, the bacterial protein target of streptomycin^36,37^ and (2) the acquisition of streptomycin resistance *via* transposition of the streptomycin resistance gene pair strA/strB in the transposon Tn5393 on the nonconjugative plasmid pEA29^37–39^. Genome resequencing can provide additional means beside PCR based genotyping to track the prevalence and spread of Streptomycin-resistant (SmR) strains in commercial orchards.

We have performed a scan of genome-wide single nucleotide polymorphisms (SNPs) and short insertion/deletions (Indels) across chromosomes and plasmids, and have identified highly polymorphic regions across the genome of 41 geographically diverse EA strains. Our analysis reports distinct sub-population structure and the role of purifying and balancing selection on genetic diversity and structure in EA strains.

## Materials and Methods

### Sample collection and strain culture

Total 41 strains were used for genome resequencing analysis (Table 1; Supplementary Dataset S1). A set of 30 strains were obtained from Dr. Steve Beer’s collection at Plant Pathology and Plant-Microbe Biology, Cornell University. One strain, ‘ZYRKD3-1’ (a deletion mutant of AvrRpt2 effector in Ea1189 strain), was obtained from Dr. Frank Zhao at University of Illinois, Urbana-Champaign. In addition, we have isolated 5 and 3 new EA strains from fire blight infected samples received from commercial orchards in Washington State and New York State in 2018, respectively. Overall, this collection had 9 Canadian strains from Ontario, Saskatchewan, Quebec, and Alberta regions, 30 USA strains from Virginia, Idaho, Wisconsin, New York, California, Minnesota, Michigan, Illinois, Washington, Georgia, and Texas regions, 1 ‘CFBP 1430’ strain from France^40^, and an ‘ZYRKD3-1’ mutant of Ea1189 strain from Germany^18,41^. These EA strains belonged to several different host plants including apple, pear, plum, crabapple, raspberry, cotoneaster, amelanchier, sorbus, blackberry, hawthorne, raphiolepsis, photinia, and mayhaw (Table 1; Supplementary Dataset S1).

**Table 1:** Summary of various *Erwinia amylovora* (EA) strains analyzed in this study.

S No.	EA Strain	Strain Identifier	Another Identifier	Place	Country	Host	Plasmid/s	Group	Comments	Reference
1	EaNY2018a	Base1	EaBase1	New York	USA	Apple	pEA29	G1		This Study
2	CFBP1430	CFBP1430	CFBP1430	Lille	France	Crataegeus	pEA29	G1		Smits *et al*.^13^
3	EaNY2018b	NY1	EaNY1	New York	USA	Apple	pEA29	G1		This Study
4	EaNY2018c	RJO001	EaRJO001	New York	USA	Apple	pEA29, pEA72	G1		This Study
5	EaWA2018a	WA1	EaWA1	Washington	USA	Pear	pEA29	G1		This Study
6	EaWA2018b	WA2	EaWA2	Washington	USA	Pear	pEA29	G1		This Study
7	EaWA2018c	WA3	EaWA3	Washington	USA	Crabapple	pEA29	G1		This Study
8	EaWA2018d	WA7	EaWA7	Washington	USA	Pear	pEA29	G1		This Study
9	EaWA2018e	WA8	EaWA8	Washington	USA	Pear	pEA29	G1		This Study
10	Ea114	114	YUBA 2	California	USA	Pear	pEA29, pEA72, pEA3	G1	Streptomycin Resistance	
11	Ea235	235	1548B	New York	USA	Pear	pEA29	G1		
12	Ea245	245	PEAR #1	Illinois	USA	Pear	pEA29	G1		
13	**Ea247r1**	247	1273	Washington	USA	Pear	pEA29, pEA72	G1	Streptomycin Resistance	
14	**Ea247r2**	247	1273	Washington	USA	Pear	pEA29, pEA72	G1	Streptomycin Resistance	
15	**Ea265r1**	265	E2002A	Ontario	Canada	Apple	pEA29	G1		Norelli *et al*.^60^
16	**Ea265r2**	265	E2002A	Ontario	Canada	Apple	pEA29	G1		Norelli *et al*.^60^
17	**Ea266r1**	266	E4001A	Ontario	Canada	Apple	pEA29	G1		Norelli *et al*.^60^
18	**Ea266r2**	266	E4001A	Ontario	Canada	Apple	pEA29	G1		Norelli *et al*.^60^
19	Ea267	267	E4003P	Ontario	Canada	Pear	pEA29	G1		Norelli *et al*.^60^
20	Ea269	269	E7001M	Saskatchewan	Canada	Crabapple	pEA29	G1		Norelli *et al*.^60^
21	Ea271	271	E7003M		Canada	Sorbus	pEA29	G1		
22	Ea272	272	E7004M	Saskatoon	Canada	Amelanchier	pEA29	G1		
23	**Ea273r1**	273	273	New York	USA	Apple	pEA29, pEA72	G1		Norelli *et al*.^60^
24	**Ea273r2**	273	273	New York	USA	Apple	pEA29, pEA72	G1		Norelli *et al*.^60^
25	Ea284	284	137wt	Michigan	USA	Crabapple	pEA29	G1		Norelli *et al*.^60^
26	Ea359	359	CRAT.1	New York	USA	Crataegeus	pEA29	G2		
27	Ea44	401		New York	USA	Cotoneaster	pEA29	G2		
28	Ea470	470	1	New York	USA	Crabapple	pEA29	G1		
29	Ea472	472	6	New York	USA	Hawthorne	pEA29	G2		
30	Ea478	478		New York	USA	Sorbus	pEA29	G2		
31	Ea514	514	Eabb76	Illinois	USA	Blackberry	pEA29	G2		
32	Ea525	525	BB2,AFRS130	Illinois	USA	Blackberry	pEA29	G2		
33	Ea526	526	AFRS601	Wisconsin	USA	Raspberry	pEA29	G1		
34	Ea533	533	BR89 FR41;AFRS105	Alberta	Canada	Amelanchier	pEA29	G3		
35	Ea548	548		Texas	USA	Apple	pEA29	G1		
36	Ea552	552	101	Georgia	USA	Mayhaw	pEA29	G2		
37	Ea570	570	24	California	USA	Cotoneaster	pEA29	G2		
38	Ea571	571	77	California	USA	Photinia	pEA29	G2		
39	Ea572	572	444	California	USA	Raphiolepsis	pEA29	G2		
40	Ea586	586	FB 93-1	Idaho	USA	Apple	pEA29	G1		
41	Ea588	588	PFB-5	Idaho	USA	Plum	pEA29	G1		
42	Ea600	600	AFRS451	Virginia	USA	Asian pear	pEA29	G1		
43	Ea624a	624a	4-96a		Canada	Raspberry	pEA29	G3		
44	Ea646	646	5	Quebec	Canada	Raspberry	pEA29	G3	—	
45	**ZYRKD3-1r1**	1189			Germany	Apple	pEA29	G1	AvrRpt2 mutant (Ea1189)	Zhao *et al*.^18^
46	**ZYRKD3-1r2**	1189			Germany	Apple	pEA29	G1	AvrRpt2 mutant (Ea1189)	Zhao *et al*.^18^

The three distinct sub-groups identified in EA strains are listed as G1 (group 1), G2 (group 2), and G3 (group 3). The strain names in bold were sequences with two replicates. The ‘r1’ and ‘r2’ letters after five strains indicated the two technical replicates for the corresponding strains.

For strain isolation, fire blight infected twigs were collected and saved in a plastic zip-top bags with a paper towel. The tissue samples were stored at 4 °C until bacterial strain isolation. The sample tissues were surface sterilized using 70% ethanol and 50% bleach and were dissected into 1-inch samples. Bark was removed from the infected twigs with a pruning scalpel. The remaining shoot was cut into 4–6 slices of the cambium by avoiding the pith. The slices were placed in ethanol for 1 minute and transferred to 50% bleach for 5–10 minutes. The tweezers were sterilized during procedure. The cambium slices were cleaned two times with E-pure water for 1 minute and soaked into E-pure sterile water for 1 hour. The clean samples were placed on sterile paper towels for drying. The bacteria were grown by placing the cleaned samples on a petri dish containing the Kings B (KB) media. The culture plates were sealed with parafilm and incubated at 27–29 °C for 1–2 days. A sterile loop was used to pick up single colonies of newly collected strains and old strains and streaked onto a new plate containing LB agar media. The plates were incubated at 29 °C for 1–2 days to grow pure strain cultures for DNA extraction.

### DNA extraction, library preparation, genome sequencing

Genomic DNA was extracted using Wizard Genomic DNA Purification Kit from Promega according to the manufacturer’s protocol. In brief, single cell bacterial colonies were grown overnight from each strain. Total 1 ml of 20 hours overnight grown culture was transferred to the 1.5 ml centrifuge tube. The tube was centrifuged at 13,000 g for 2 minutes to pellet cells. Supernatant was discarded and 600 μl of nuclei lysis solution was added by gentle mixing. Samples were incubated at 80 °C for 5 minutes, cooled to room temperature, and 3 μl of RNase solution was added. Samples were gently inverted few times for mixing well and incubated at 37 °C for 45 minutes. After cooling to room temperature, 200 μl protein precipitation solution was added to cell lysate and vigorously vortexed for 20 seconds at high speed. Samples were placed on ice for 5 minutes and centrifuged at 13,000 g for 3 minutes. DNA containing supernatant was transferred to a clean 1.5 ml tube with 600 μl of isopropanol. Samples were gently mixed by inverting the tubes. DNA was precipitated by centrifuging at 13,000 g for 2 minutes and washed using 600 μl of 70% ethanol by repeating as above. Supernatant was discarded and DNA pellet was air-dried for 15 minutes. The pellet was eluted in 100 μl of DNA rehydration solution at room temperature overnight. The DNA quality was assessed using 1% agarose gel electrophoresis and quantified with Nanodrop^TM^ One/OneC Microvolume UV-Vis Spectrophotometer (Thermo Fisher Scientific, USA).

Total 50 ng DNA was used to prepare genome sequencing libraries using Illumina Nextera skim sequencing library preps at Institute of Biotechnology, Cornell University, Ithaca, NY. Library quality and quantity was checked with Agilent Bioanalyzer (Agilent; www.agilent.com). Samples from individual bacterial strain were barcoded and whole genome sequencing was performed using a single Illumina Mi-Seq lane to obtain 2 × 250 bps paired-end reads.

### Sequence analysis and variant discovery

Barcode sequences were used to separate individual samples to use for quality analysis with fastqc program (http://www.bioinformatics.babraham.ac.uk/projects/fastqc/). Sequencing adaptors and low-quality sequences at read ends were trimmed using Trimmomatic software^42^ with LEADING:20, TRAILING:20, SLIDINGWINDOW:4:15, AVGQUAL:20, and MINLEN:25 parameters. The reads with a quality score below the threshold of 20 were removed from further analysis. The resulting high-quality reads were mapped against EA CFBP 1430 genome^13^ using burrows-wheeler aligner (bwa) with default parameters^43^. The mapping record was obtained as sequence alignment/map format^44^ by assigning unique read group ID for each sample. The alignment files were processed to remove PCR duplicated reads and sorted to obtain binary alignment format (BAM) using SAMtools^44^.

Variant analysis was performed with Genome Analysis Toolkit (GATK version 3.8.0)^45^ using parameters as; -ploidy 1 -stand_call_conf 30 -variant_index_type LINEAR -variant_index_parameter 128000 to obtain SNPs and short Indels across the EA genome. The BAM files were processed to generate genotype variant call format (gVCF) files for each strain separately with HaplotypeCaller plugin in GATK. The gVCF files were used to run GenotypeGVCF module to obtain a single VCF file for Erwinia population. The variants were separated into SNPs and Indels for quality filtration. SNPs were filtered using VariantFilteration plugin with parameters “QD < 2.0||FS > 60.0||MQ < 40.0||MQRankSum < −12.5||ReadPosRankSum < −8.0”. Indels were filtered with parameters “QD < 2.0||FS > 200.0”. The resulting variant datasets were used as base calibration to repeat the variant analysis as above. The base calibration analysis helps eliminate false positives due to several factors associated with library preparation and sequencing. The final set of recalibrated SNPs and Indels was filtered further to retain variants present in at least 90% of the population and had mean read depth score of 3 or more. The resulting SNP dataset was used for diversity and population genetic structure analysis.

A similar analysis was performed using pEA29, pEA72, pEAE2, pEI70, pEA3, pEAR4.3, and pEAR5.2 EA plasmid sequences as reference. The plasmid sequences were obtained from an NCBI genome search using “*Erwinia amylovora*” as the keyword. The reads were separately aligned against these plasmid reference sequences to generate SNPs and short Indels. The variants in “pEA29” across all the strains were annotated using the annotation information available in NCBI for this plasmid. Variants were also analyzed across virulence-related thiamine biosynthesis operon and other putative genes in the ubiquitous pEA29 plasmid^28^.

Variants were annotated using ANNOVAR program^46^ as per the CFBP 1430 coding gene information. SNPs were annotated for Intergenic, Upstream and downstream (2 Kbs upstream and downstream from the transcription start site), 5′UTR and 3′UTR, Intronic, exonic, and splicing sites. The SNPs in exonic regions were further characterized into synonymous (no amino acid change) and nonsynonymous (amino acid change) mutations. Exonic indels were characterized for frameshift mutations.

### Population genetic analysis

Genome-wide statistics for variant distribution, nucleotide diversity (π), TajimaD, and fixation index (Fst) statistics were computed using the VCFtools software^47^. The population structure in EA was determined with three different methods using the SNP dataset. First, a principal component analysis (PCA) was conducted using Tassel v5^48^ and a biplot between first and second principal components was used to determine the structure. Second, the SNPs were used to obtain an identity-by-state distance matrix using PLINK v1.07^49^ and a neighbor-joining tree was visualized using the distance matrix in MEGA7 software. Third, the fastSTRUCTURE software^50^ was used to cluster Erwinia strains with a prior run using 1 to 10 subgroups (K = 1 to 10). The “choosing model complexity” script was used to obtain best sub-cluster model in Erwinia population. The cluster membership for each strain was determined with 1000 permutations. To further assess the role of selection pressure on genome differentiation, we computed the nucleotide diversity, TajimaD and fixation index statistics separately across the Erwinia sub-populations using 5 kbs genomic windows with vcftools v0.1.13 software^47^.

## Results

### Patterns of genomic variation in E. amylovora

**Figure 1:**
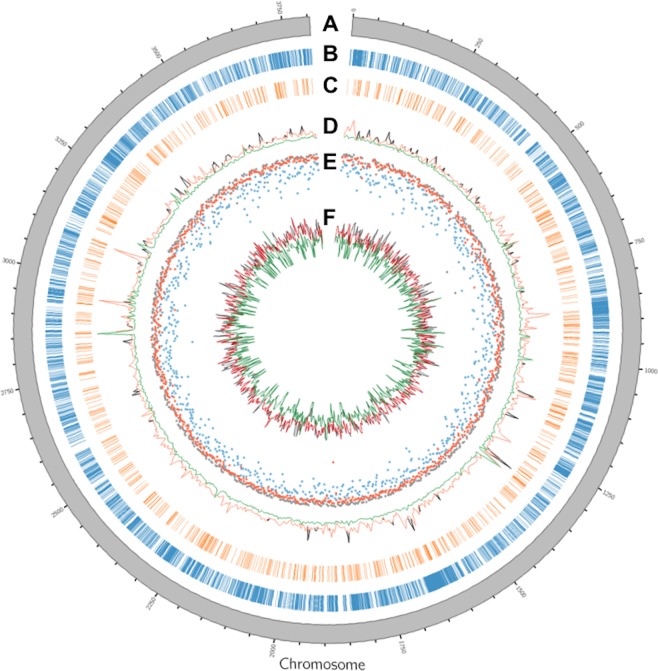
Circos plot showing the distribution of various genomic features across *Erwinia amylovora* (EA) chromosome. (**A**) EA chromosome in megabases; (**B**) Genome-wide distribution of identified single nucleotide polymorphisms (SNPs); (**C**) Genome-wide distribution of identified insertions/deletions (Indels); (**D**) Patterns of nucleotide diversity (π) in G1 (black), G2 (orange), and G3 (green) as estimated using 5 kilobase genomic window scans; (**E**) Fixation index (Fst) estimates between strains from G1-G2 (blue), G1-G3 (grey), and G2-G3 (red) inferred using 5 kilobase genomic window scans; (**F**) TajimaD values obtained using 5 kilobase genomic window scans in G1 (grey), G2 (red), and G3 (green).

A total of 46 samples from 41 EA strains were sequenced in this study (Table 1), generating about 11.6 million sequences and representing 16.5X genome coverage of the entire ~3.8 megabases *E. amylovora* genome (Supplementary Dataset S1). After eliminating the 2.36% of low quality read sequences, the genome coverage dropped to 16.1X, ranging from 3 to 36X per sample (Supplementary Dataset S1). The percentage of reads aligned to the reference genome ranged from 86% to 99.6% with an average alignment rate of 95.7%. We replicated 5 strains to calculate any variant discrepancies due to strain isolation, library construction, and sequencing analysis. Variant analysis across the EA chromosome (Fig. 1A) identified a total of 72,741 SNPs (Fig. 1B) and 2,500 Indels (Fig. 1C) in 41 EA strains with an average nucleotide diversity of 0.13. Replicated strains displayed highly consistent variation patterns, and variation within replicates ranged from 0.25% to 1.65% for ZYRKD3-1 and Ea265, respectively (Supplementary Dataset S1). In addition, Sanger sequencing of five representative polymorphisms between three different strains confirmed the presence of all identified SNPs at detected genomic locations. Variant annotation using CFBP 1430 coding sequence information found that 72,741 loci represented a total of 73,382 alternate SNP alleles in the population, from which 47,869 were transitions and 25,513 were transversions. About 78.7% (n = 57,816) of these SNP alleles were located in the exonic sequences followed by 21.1% (n = 15,535) in gene upstream-downstream regions. The remaining 31 SNP variants were present in ncRNA exonic regions. Further annotation of the 78.7% of coding region SNPs showed that 32.6% of them were nonsynonymous while 65.4% were synonymous mutations. Remaining SNPs in coding regions either represent stop gain (n = 234), stop loss (n = 44), or unknown (1.4%) mutations. The ratio of nonsynonymous to synonymous mutations (Ka/Ks) was 0.49. However, the average genome-wide Ka/Ks ratio provides little information about particular genomic regions under positive, neutral, or purifying selection. Thus, we calculated the nonsynonymous and synonymous mutations within 2 kbs genomic windows across the EA genome. This analysis revealed that about 10.5% of the EA genome was under positive selection (Ka/Ks > 1), 5.8% under neutral selection (Ka/Ks = 1), and remaining 83.7% showed purifying selection with Ka/Ks < 1 (Supplementary Dataset S2).

Annotation of Indels represented 2,521 alternate alleles distributed as 1,054 exonic, 1,459 upstream-downstream, 6 ncRNA exonic, and 2 UTR-5′ variants. The exonic Indels had 40.7% frameshift deletions, 32.9% frameshift insertions, and approximately 20% nonframeshift Indels. There were also 13 and 10 Indel mutations associated with gain or loss of stop codon.

### Variations in CRISPR, effectors, and streptomycin resistance genomic regions

**Figure 2:**
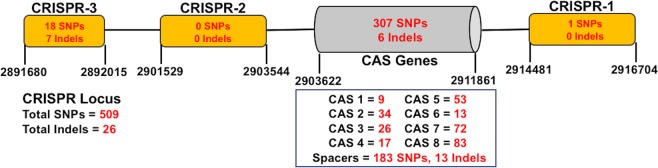
Diagram showing organization of clustered regularly interspaced short palindromic repeat (CRISPR) locus in CFBP 1430 *Erwinia amylovora* genome. The yellow boxes represent the three CRISPR regions and the grey box represents the genomic region related to CRISPR-associated (CAS) genes within CRISPR locus. The number of detected variants are with red font.

A targeted genomic analysis identified a total of 509 SNPs and 26 Indels across the CRISPR locus in CFBP 1430 genome (Fig. 2). About 60.3% (n = 307) of SNPs and 6 Indels were present on the CRISPR-associated (CAS) gene sequences. The number of SNPs ranged from 9 to 83 on different CAS genes. CRISPR 1 and CRISPR 2 were the least variable sequences on this locus. CRISPR 1 contained one SNP and no Indel, while CRISPR 2 had no SNPs or Indels. In contrast, the smallest CRISPR 3 region was more variable, with 18 SNPs and 7 Indels. The remaining 183 SNPs and 13 Indels were located on the spacer sequences of the CRISPR locus.

Similar analysis detailed polymorphism patterns across type III secretion system (T3SS) effectors on CFBP 1430 genome^13^. A total of 660 SNPs were present in Hrp T3SS locus harboring 27 genes, 732 SNPs in the PAI-2 inv/spa-type T3SS, and 1,062 in the PAI-3 inv/spa-type T3SS regions on Erwinia genome (Supplementary Dataset S3). The singleton eop2, HopPtoC, and AvrRpt2 effector genes had 54, 39, and 5 SNPs, respectively. The mutations in the AvrRpt2 effector were identified in strains Ea478, Ea514, Ea525, Ea526, Ea533, Ea624a, and Ea646. These mutations were different from a previously studied mutation causing cys156 to ser156 amino acid change in the AvrRpt2 effector mutant^19,20^. However, comparison of amino acid sequences suggest that all five mutations cause amino acid changes in the translation frame (Supplementary Dataset S4). A previously generated AvrRpt2 effector deletion mutant strain ‘ZYRKD3-1’ was also included in this study^18^. However, the short read sequencing approach used here was not able to identify the large insertional sequences reported for ‘ZYRKD3-1’.

The Erwinia collection used here contained two strains, Ea144 and Ea247, with a point mutation on the *rpsL* gene associated with streptomycin resistance^36,37^. A single SNP (T to C substitution) at 3,491,048 position at the *rpsL* gene on CFBP 1430 genome was detected only in strains Ea144 and Ea247, which causes the known lysine to arginine (K/R) substitution for streptomycin resistance (Supplementary Fig. S1). The *rpsL* gene also harbored two other mutations at positions 3,490,942 and 3,490,960 in Ea533, Ea624a, and Ea646, although these mutations did not translate into a different amino acid or changes in open reading frame.

### Population structure and divergence between E. amylovora strains

**Figure 3:**
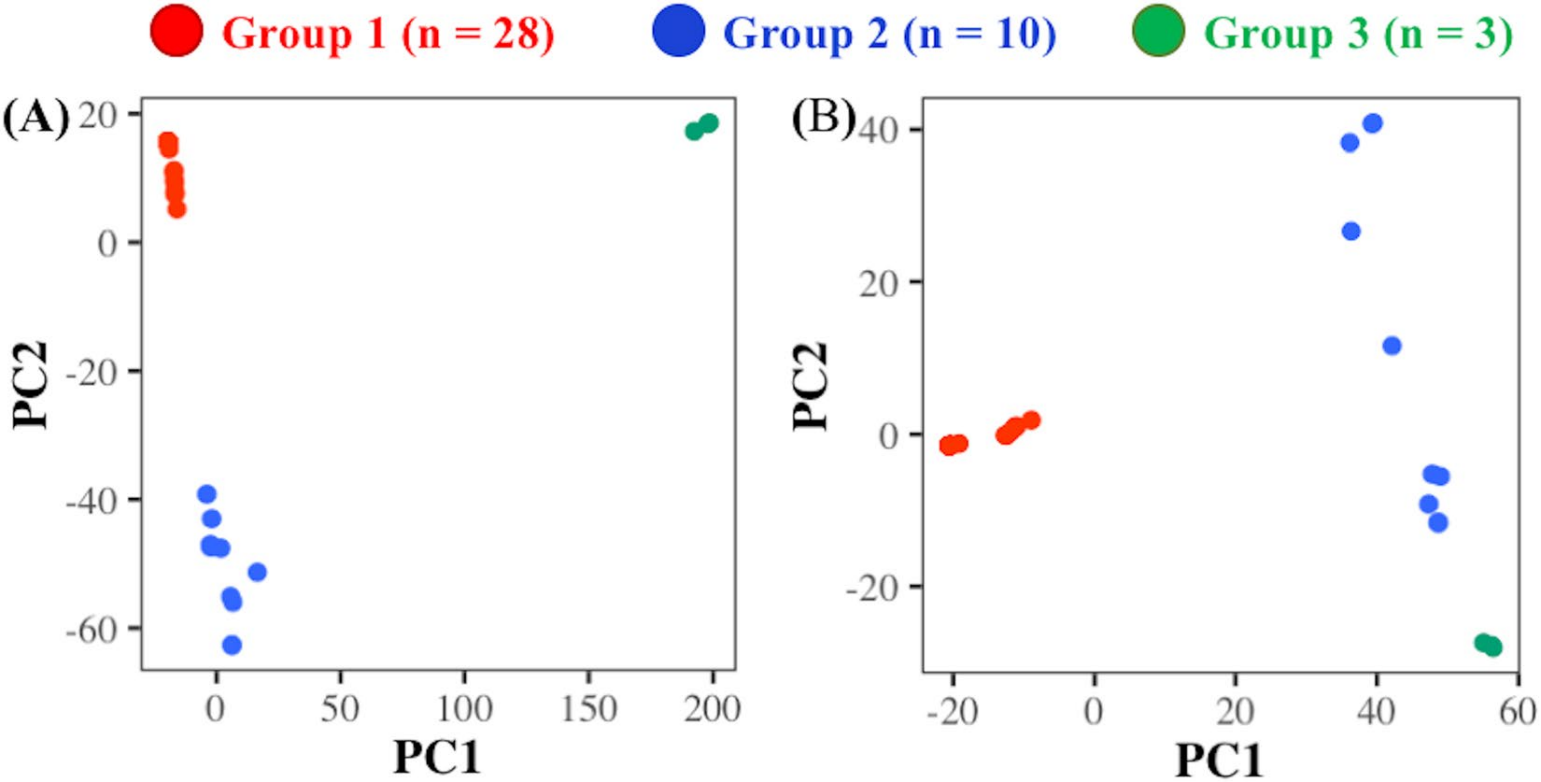
Biplots between the first two components (PC1 and PC2) from principal component analysis of chromosomal SNPs from 41 *Erwinia amylovora* strains before (**A**) and after (**B**) minor allele filtering from the variant dataset.

We used all 72,741 SNPs to determine the structure in Erwinia strains using PCA. A biplot between the first two principal components (PCs) identified three distinct clusters of the Erwinia strains (Fig. 3A). The three population sub-groups consisted of twenty-eight (group 1; G1), ten (group 2; G2), and three (group 3; G3) strains, respectively (Supplementary Dataset S5). G1 contains Erwinia strains from Canada, USA, Germany, and France with widespread hosts including pear, apple, crabapple, sorbus, amelanchier, raspberry, and plum (Supplementary Dataset S5). In contrast, G2 mainly represents USA strains from New York, Illinois, Georgia, and California. These strains were collected on host plants from cotoneaster, crataegeus, sorbus, blackberry, photinia, and raphiolepsis. The three strains Ea646, Ea533, and Ea624a forming G3 were mainly from Canada and were collected from amelanchier and raspberry hosts.

A genome-wide TajimaD estimate for the EA was −1.53, indicating an excessive presence of rare alleles in the population. Distinct sub-grouping in Erwinia strains can partially explain the high proportion of rare alleles where certain variants can only exist in a specific sub-group. At the same time, the rare alleles in EA strains can also drive the sub-clustering pattern observed from PCA analysis. To test whether rare SNP alleles influence population structure, we performed the PCA analysis using 10,250 filtered SNPs with a minor allele frequency (MAF) threshold of ≥0.1. The Erwinia strains still appeared to have three sub-groups, but the distinction between G2 and G3 was less clear (Fig. 3B). The EA strains in G2 showed a more dispersed pattern after removing minor allele variants, but the effect was much less in G1 and G3 (Fig. 3B).

**Figure 4:**
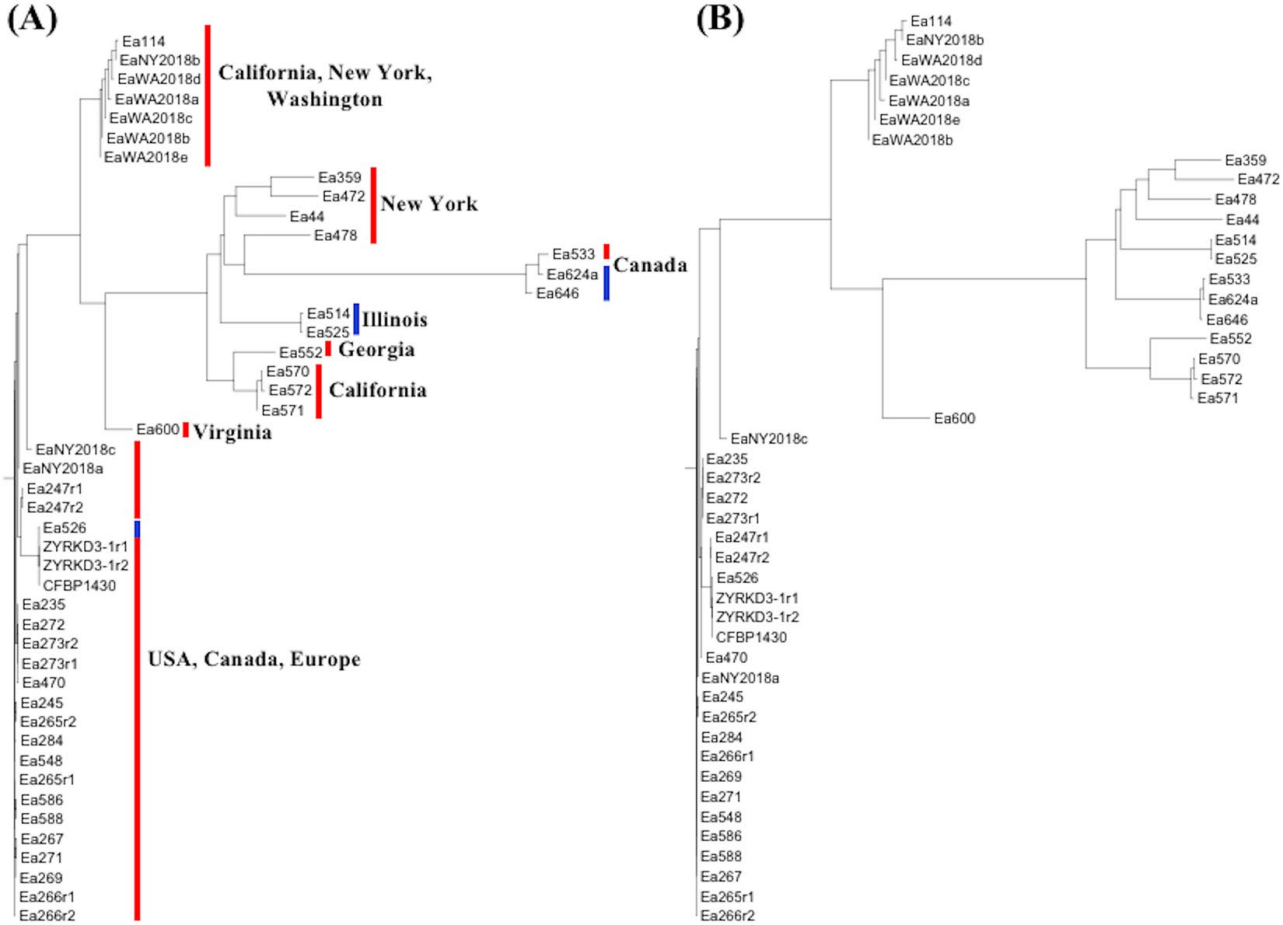
Phylogenetic trees constructed using high-quality chromosomal SNPs to determine the relationships between different *Erwinia amylovora* strains before (**A**) and after (**B**) minor allele filtering from the variant dataset. The ‘r1’ and ‘r2’ letters after five strains indicated the two technical replicates for the corresponding strains.

We further analyzed the phylogeny of 41 Erwinia strains using a sub-set of 2,017 high quality SNPs with average read depth ≥6 to generate a distance matrix and neighbor-joining (NJ) tree. The co-localization of the replicated samples supports the reliability of skim sequencing for small bacterial genomic analysis (Fig. 4). Phylogenetic analysis also confirmed the sub-groups in EA strains, where three strains in G3 appeared to be distantly related to strains from other two groups (Fig. 4A). Similarly, phylogeny construction using MAF filtered SNPs also resulted in consistent relationships between Erwinia strains, but the level of similarity increased between strains from G2 and G3 (Fig. 4B). A closer analysis of the phylogenetic tree identified several aspects of geographical spread and host specificity in highly variable EA strains. For example, the three most distant Canadian strains in G3 (Fig. 3A) showed immediate clustering with the four New York strains (Ea359, Ea472, Ea44, Ea478) in G2. The remaining G2 strains showed sub-clustering according to different US states. For example, there was a sub-group of 2 strains, Ea514 and Ea472, from Illinois. A single strain from Georgia was grouped on a sub-node shared with three California strains (Ea570, Ea571, Ea572). The strain Ea600 was partitioned into a totally separate node from the above strains. The least diverse G1 strains formed two sub-trees, one consisting of 7 strains from California, New York, and Washington, and the second of many strains from USA, Canada, and Europe (Fig. 4). The two European strains, ‘CFBP1430’ and ‘ZYRKD3-1’, were co-localized on the same node with a USA strain, Ea526, from Wisconsin. The five RI strains showed different clustering patterns in this study. The 2 RI strains from Illinois formed a distinct group, while 2 RI strains from Canada (Ea624a, Ea646) were grouped along with the SI strain Ea533. The remaining RI strain (Ea526) was clustered with the SI strains in G1.

**Figure 5:**
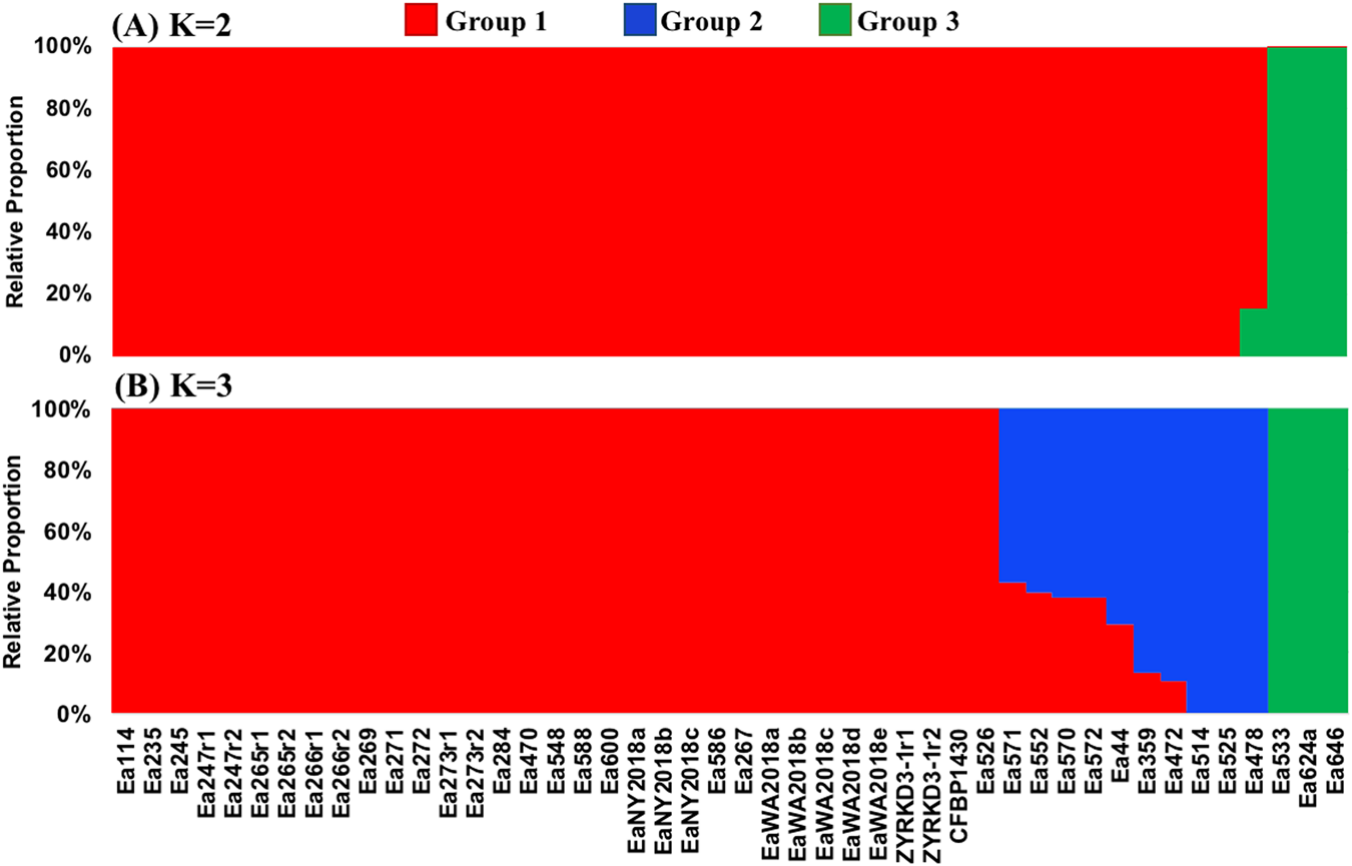
Population structure and genome admixture between different *Erwinia amylovora* strains as inferred from Bayesian analysis using K = 2 (**A**) and K = 3 (**B**). The ‘r1’ and ‘r2’ letters after five strains indicated the two technical replicates for the corresponding strains.

The phylogenetic pattern of Erwinia strains further extended to host specificity. For instance, G2 and G3 strains belonged to host plants from crataegeus, cotoneaster, sorbus, photinia, raphiolepsis, amelanchier, blackberry, and raspberry (Supplementary Dataset S5). In contrast, most G1 strains were isolated from host plants including apple, pear, crabapple, and plum. Only 3 strains in G1 belonged to host plants from sorbus, amelanchier, and raspberry (Supplementary Dataset S5). Analysis of population admixture also revealed three main groups in the population (Fig. 5). Some strains have clearly distinct genome compositions from one specific group. In contrast, few G2 strains including Ea570, Ea571, Ea572, Ea552, Ea44, Ea359, and Ea472 had genome admixture from G1 strains (Fig. 5).

### Distribution of genomic variability in E. amylovora sub-populations

**Figure 6:**
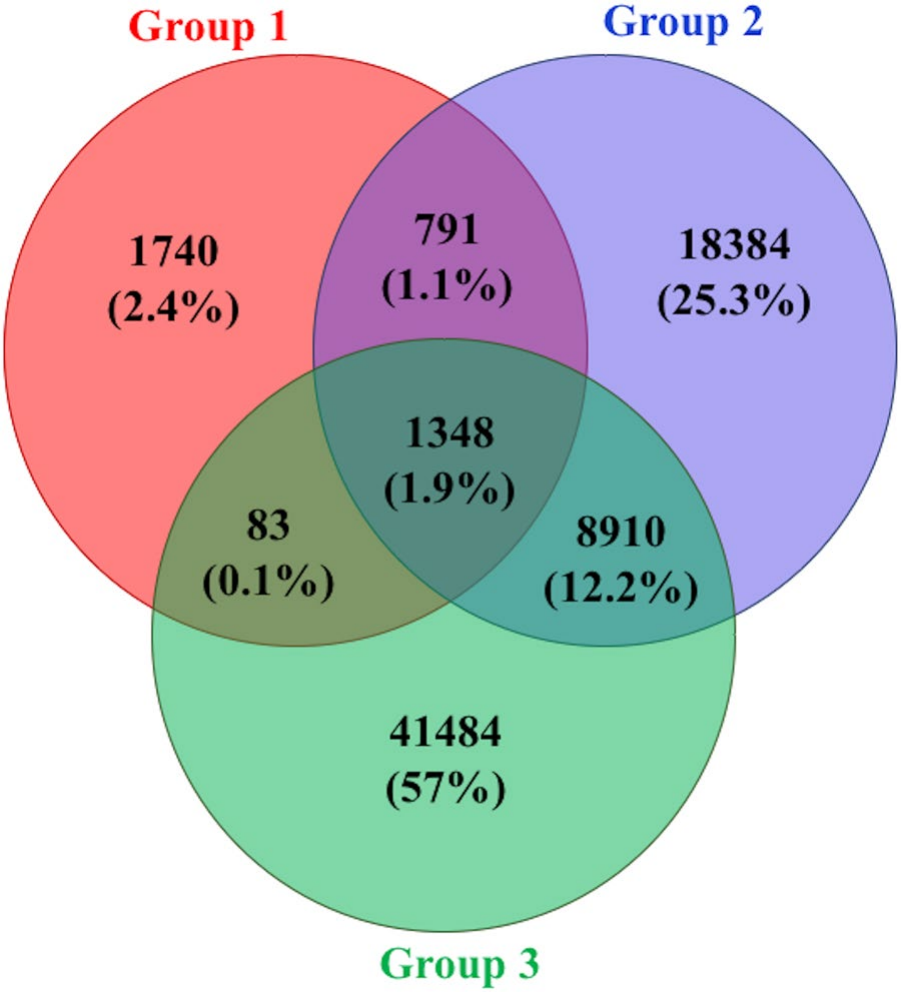
Venn diagram showing distribution of unique and common SNPs between three different sub-groups in *Erwinia amylovora* strains.

A sub-population variant analysis further clarified the genomic diversity between and among the three EA strain groups. For instance, each sub-group had a large proportion of unique SNPs and only 1.9% of the total identified SNPs were shared between them (Fig. 6). Although G3 appeared to have the highest number of unique SNPs (Fig. 6), use of a reference sequence for alignment and SNP calling can significantly influence these results. A reference genome from G3 could identify a smaller number of unique SNPs in this cluster than using G1 strain CFBP 1430 strain as a reference. Thus, we used nucleotide diversity as a measure to evaluate the differences between each sub-group. The level of genetic diversity was highest in G2 (π_G2_ = 2.3 × 10^−3^) followed by G3 (π_G3_ = 7.9 × 10^−4^). G1 exhibited the least amount of diversity with π_G1_ = 1.9 × 10^−4^. These trends also remained consistent after accounting for sample size within each group. On average, G1 had about 120 SNPs per strain, while G2 and G3 had 2773 and 1859 average SNPs per strain, respectively. About 51.2% (n = 37,268) of total SNPs were identified from inter-group diversity analysis, while the remaining 48.8% SNPs were specific to inter-group comparisons.

Nucleotide diversity analysis showed that approximately 81% of the Erwinia genome had at least a five-fold difference in nucleotide diversity between G2 and G1 (π_G2_/π_G1_), which decreased to 31.1% between G2 and G3 (π_G2_/π_G3_) (Fig. 1D; Supplementary Dataset S6). The weighted fixation index (Fst) values were 0.63 and 0.60 after comparing G2 with G1 and G3, respectively. About 81.4% of the Erwinia genome had Fst values more than 0.5 between G2 and G1 (Fig. 1E). The percent of Fst values greater than 0.5 increased to 98% when G3 was compared with the other two groups. Many highly diverse genomic regions had negative TajimaD measures in G1 and positive TajimaD measures in G3 (Fig. 1F; Supplementary Dataset S6).

Strains from G1 mainly infect pear, apple, and crabapple, hence variant distribution in the effector regions of G1 strains can provide important clues about strain virulence. We filtered hypothetical protein genes and analyzed variant frequency in remaining genes in the effector loci. The inv/spa-type T3SS (PAI-3) effector loci exhibited highest variation, with a total of 89 variants distributed across 16 genes (Supplementary Dataset S3). The genes for T3SS components PulD and EscV were among the highly variable genes at this locus. In comparison, the Hrp T3SS and inv/spa-type T3SS (PAI-3) effectors only had 22 and 20 variants distributed across total 30 and 17 genes, respectively. Approximately 40.9% of genes within the T3SS effector loci did not show any polymorphism between G1 strains and 73.4% (n = 39) of the remaining SNP containing genes had at least one nonsynonymous substitution, which can cause an amino acid change in a protein (Supplementary Dataset S3). A single stopgain mutation was located in the HopPtoC effector gene at 835,315 position and was only present in the Ea600 strain.

### Plasmid sequence variation patterns in E. amylovora

Sequence alignments were obtained in three out of seven reference plasmids in at least a single EA strain (Table 1). The non-conjugative plasmid pEA29 was present in all 41 Erwinia strains. Another plasmid, pEA72, was detected in four strains: Ea114, Ea247, Ea273, and EaNY2018c, while pEA3 was present in the single strain Ea114. The ubiquitous pEA29 plasmid had a total of 649 variants and average nucleotide diversity (π_P_) of 0.15. PCA and phylogenetic analysis using plasmid variants indicated almost similar population structure patterns as observed from genomic variants. Three G3 strains formed a clearly separated group from G1 and G2 strains (Supplementary Fig. S2). In addition, the presence of rare alleles in plasmid sequence also influenced the population structure in EA and the G2 and G3 strains showed co-localization on the PCA biplot and NJ tree after filtering minor alleles from the population (Supplementary Fig. S2B). Interestingly, G1 strains were split into two distinct groups (Supplementary Fig. S2A) and the separation of G1 strains were more prominent after minor allele filtration (Supplementary Fig. S2B) in both PCA and phylogenetic analysis. It appeared that plasmids of recently obtained strains from New York and Washington along with Ea600 and Ea114 had notable differences than the remaining G1 strains (Supplementary Fig. S2B; Dataset S7). These strains had pear as host plant except EaNY2018b and EaWA2018c that were obtained from apple and crabapple hosts (Supplementary Dataset S7).

Three thiamine biosynthesis genes (thiozole biosynthesis, thiozole synthase, sulphur carrier) and one thiamine pyrophosphate riboswitch had total 32 variants in all the strains. The number of variants across other putative virulence related genes varied from 1 to 26. Level of variation in virulence-related genes of pEA29 also accompanied the sub-grouping observed in EA strains, and G2 strains had highest plasmid variation across these regions.

## Discussion

Three distinct population sub-groups (G1, G2, G3) were determined from the chromosome and plasmid sequence variants in EA strains. Geographical isolation appears to define the separation of G2 from G3 strains, and also the sub-groups within G2 cluster. All three G3 strains were from Canada, while strains from different U.S. states appear on separate sub-nodes in G2 group. Since they were obtained mainly from wild hosts, G2 and G3 strains have probably been evolving independently in their respective geographical regions, and chances of spread between regions through material transfer and other means is unlikely. Previous studies have established that EA originated in eastern North America and later spread across the continent and to other countries^21,25,51^. The highest genetic diversity of G2 strains, as expected at the center of origin^52^, suggests that geographical regions corresponding to these strains might represent the EA center of origin. Partial similarity in genomic composition of G2 and commercially relevant G1 strains further suggests the latter might have disseminated from the original G2 strains. Recently, eastern U.S. has been proposed as the EA origin^25^, which, collectively with the results from this study, suggests that G2 strains from New York most likely represent the EA center of origin. The New York strains showed diffusion either to Canada or various geographical locations in USA, including Illinois, Georgia, and California (Fig. 4). The G1 strains were either directly disseminated from the New York strains or may have been selected from the remaining G2 strains.

In contrast to G2 and G3, geographical sub-grouping was not apparent in the G1 strains from different parts of U.S., Canada, and Europe. However, compared to G2/G3 groups, G1 strains reflect some differences in host specificity of EA strains. The G2/G3 groups mainly contained strains from wild hosts, but G1 mostly represents strains from apple and pear commercial orchards and very few strains from wild hosts (Fig. 3). These latter G1 strains could have been originally established on the wild plants or were dispersed from apple and pear production areas to the wild habitats. There is an indication that EA was originally present in wild hosts and later spread to apple and pear production areas^6^, yet the chances of cross-contamination from cultivated to wild habitats cannot be ignored. Overall, the results suggested a limited host specificity in EA strains within G1. Host specificity has earlier been determined between EA strains from SI and RI hosts^7,24,25^, but our results also showed some inconsistencies from previous reports. For instance, one RI strain clustered with SI strains in G1 while two RI and one SI strains formed the G3 cluster. We must specify that the RI strains used in the current study are different from the previous ones^24,25^, which can explain the inconsistencies between these studies. Some of these inconsistencies can also be attributed to the approaches used for phylogeny construction. For example, errors inherent in the short-read sequencing technologies^53^ can create bias in phylogenetic relationships from the variant datasets. However, high consistency between the replicated strains, confirmation of detected variants using Sanger sequencing, and identification of previously known mutations supports the reliability of variants used in this study. Therefore, we expect that sequencing and comparison of more RI strains against SI strains can clarify the distinction between these two groups. The SI and RI strains further differentiate based on the presence or absence of a sorbitol operon and impairment of the PrtA secretion system^54^. However, a reference-based alignment of short reads provides a less suitable approach to detecting large insertions/deletions in the aligned genomes. A genome assembly approach combining long read from PacBio and Nanopore with the short Illumina sequencing reads can highlight such differences in SI and RI strains^24,25^.

Phylogenetic analysis also identified two less distinct groups within G1 strains. The recently collected strains from Washington (EaWA2018a, EaWA2018b, EaWA2018c, EaWA2018d, EaWA2018e) and New York (EaNY2018b) along with an earlier collected Ea114 strain from California were clustered on a separate node from the remaining G1 strains. Interestingly, the distinction was highly prominent with the plasmid variants, suggesting that plasmids are evolving faster than the chromosome sequences in these strains. It further suggests the rate of spontaneous mutations were different in the latter from the rest of G1 strains. Previous reports have indicated that the rate of occurrence of spontaneous mutations is low in EA strains and a particular European strain is capable of accumulating only 46 SNPs in 48 years^25^. The European strains were introduced from original North American center through a single bottleneck event^21,55^ by EA infected plant material^6^. The two European strains in the current study were highly similar and showed clustering with the rest of G1 strains than the recent ones from Washington and New York. Thus, the estimates of spontaneous mutation rate in the European strains might not fully represent the recent strains from Washington and New York, which probably have been going through different local selection pressure due to weather and management practices in the collection orchards. Similar will be true for the Ea600 strain from Virginia that localizes on a completely separate node from the remaining G1 strains.

The selection effects were also highlighted by the differences in nucleotide diversity and allele frequencies between three EA sub-groups. First, the three EA sub-groups accompanied large number of unique polymorphisms. Furthermore, the differentiation of G1 strains was accompanied by removal of rare alleles from the original population whereas rare alleles were present with considerable frequency in G2 and G3, and removing their effect by minor allele filtering dissipated the sub-population distinction between these two EA sub-groups. These observations underline the effect of purifying and balancing selection in determining EA population structure. Purifying selection acts to remove deleterious mutations, while balancing selection maintains the level of variation after population bottlenecks created by different selection forces^56^. The frequency of these mutations drives evolution through adaptation^56,57^, which is further affected by the nature of co-evolution between pathogens and their host plants^58^. We suppose that the distinct nature of selection observed in EA populations can most likely be attributed to the co-evolution of EA from the wild hosts to the commercial apple and pear cultivations^6^. A narrow host range might have caused the removal of deleterious mutations^57^ in the G1 strains whereas expanded wild host range in G2 and G3 might lead to maintenance of EA genetic variation to balance their co-evolution with respective strains.

Six-fold more variants were detected here than from a recent study^25^, which is also much higher than the earlier comparison of EA genomes^24,25^. For instance, comparative analysis of two sequenced EA genomes, CFBP 1430 and Ea273, showed 99.9% genome similarity^13^, but a pan-genome analysis of 12 strains and diversity analysis of 30 strains exposed higher diversity in the EA strains^24,25^. Taken together, the results from current and previous studies have extended the knowledge of the genetic diversity in EA, probably due to inclusion of new strains from different host plants and diverse geographical locations. The pan-genome of 12 EA strains further suggests relatively higher genetic diversity in RI than SI strains, and also detected variation in the effector proteins^24^ that might influence host-pathogen interactions. Our targeted genome analysis also underlined nonsynonymous substitutions in the effector regions and a stopgain mutation in HopPtoC effector gene encoding a papain-like cysteine protease^13,59^. The mutations in effector genes are specifically relevant for observing differences in virulence of strains. For example, an induced deletion and single base substitution in the AvrRpt2 effector reduced infection on immature pear fruits^18–20^. Further studies can clarify the role of nonsynonymous and stopgain mutations in the effector genes in defining virulence levels of particular Erwinia strains. Previous studies also highlighted the contributions of plasmids in shaping EA genetic diversity^24,25^. As identical plasmid contents do not necessarily confer similar phenotypes^24,60^, the genetic diversity within a single plasmid might explain some of these differences. For example, we identified considerable nucleotide variation in universal plasmid pEA29 that can facilitate further research to understand its role in plasmid-conferred virulence in EA.

Skim sequencing can also provide an effective alternative to lab-based assays for studying genetic diversity and structure, and to monitor antibiotic resistance in commercial orchards. Lab-based genotyping assays can take time in terms of primer design and running experiments to amplify only the regions of interest, not the entire genome. In such cases, low-cost skim sequencing can provide a time-effective substitute to PCR-based genotyping to assess genomic diversity patterns in several strains. However, next-generation sequence analysis requires specific computational tools and expertise that might not be ideal for all labs. Furthermore, skim sequencing is a less suitable alternative to identify large insertions/deletions in the genome, and to monitor known gene mutations like rpsL streptomycin resistance^36,37^. Targeted amplification of a single gene mutation provides a more cost and time effective approach than skim sequencing to monitor streptomycin resistance in commercial orchards. However, skim sequencing will be useful to identify additional mutations in the same or related genes causing streptomycin resistance in different EA strains. The skim sequencing will also be relevant to monitor the evolution of EA strains over time, and to study new EA strains that might cause unpredictable fire blight outbreaks in commercial orchards.

In summary, sequencing and variant analysis of 41 strains revealed comparatively much higher genetic diversity in EA than previous reports. The genetic diversity in Erwinia accompanies the sub-population structure, with North American strains keeping up the highest diversity in the population. The results also indicated that group 1 and group 3 might have differentiated from original center through purifying and balancing selection, respectively. Sequencing and analysis of additional RI strains are suggested to clarify their distinction from SI strains.

## Supplementary information

Dataset S1

Dataset S2

Dataset S3

Dataset S4

Dataset S5

Dataset S6

Dataset S7

Supplementary Figures

## Data Availability

The sequence reads generated from various *Erwinia amylovora* strains in this study have been deposited in National Center of Biotechnology Information (NCBI) short read archive (SRA) database under the project identifier PRJNA544208.
