# Supplementary material for: Distinct patterns of natural selection determine sub-population structure in the fire blight pathogen, Erwinia amylovora
Source: Sci Rep. 2019 Sep 30;9:14017. doi: 10.1038/s41598-019-50589-z (PMC6768868; doi:10.1038/s41598-019-50589-z)
Supplement: Supplementary file 4 — Dataset S4 [file 41598_2019_50589_MOESM4_ESM.docx]

**Supplementary Dataset S4 corresponding to the manuscript:**

**Distinct patterns of natural selection determine sub-population structure in the fire blight pathogen, *Erwinia amylovora***

Jugpreet Singh^1^, Awais Khan^*1^

^1^Plant Pathology and Plant-Microbe Biology Section, Cornell University, Geneva, NY, 14456, USA

*Corresponding author: Awais Khan, Email: mak427@cornell.edu, Tel: +1 315 787 2446

**Supplementary Dataset S4.** The nucleotide sequence of AvrRpt2 effector gene sequence and amino acid alterations due to five SNP mutations in it.

**Mutations in the AvrRpt2 effector sequences**

>NC_013961.1:3263335-3264003 Erwinia amylovora CFBP1430 complete genome

CTAATTTTCACTGTATAACATGGCGTGTGGCACCCGCCAT[G/A]CCAGACGCTGGTTGAATGAATCCAACGGCATGGTCAGATCCGGA[C/T]CGCGTTGCGGATCGTGAAAAATAATCGCATCGTTGGGCTTGTCTATCCCGGTCAGCACAGACATATGCCAGCTGCCGGCCGGGGTTTGCCAACCAAACATAATGGGTCCATGGCGACACAAGAGATTGCCGAGTTCATTGGCTGAAAA[C/A]TGCCTTGATTCCGGTAAACTCACTTCGGCAAGGTTTTCATTTCGCATCAGCCTGAGCACATCTTCACGTTGTTGTAGTCCTTGCGGGCCTGAAGAGTCATAGAGTTCTGGCAGCCCCAGCCGTGGGCCGCTGCTGATGCTATGGCCCAGCATTCTGGTGCAGGCATACCAGCAGCCCATTCGCTCATTCTGTTGAGACACATAAGGTACATGGTTTAAGCGAAAGCCGGCGTTACTGCGAGAGCCGGAACTGCTTCCAGAGTCGTACCCTCTGG[A/T]CTTTTTACTTTTGCCGAAACATCCGCCAAAAGATGGCGCTGCGGCCGGTAGCTGGGACCAGGGTTTGATGACATCGCCATCGCGGCTTACTTTCTCTGATTGACGAGGCTGGTGCTCGATTACGACAGGGGCGGGGGATGTGAGA[T/C]GACTGACTTTCAC

**Wild Type Nucleotide Sequence of AvrRpt2 effector**

>WT-NC_013961.1

CTAATTTTCACTGTATAACATGGCGTGTGGCACCCGCCATGCCAGACGCTGGTTGAATGAATCCAACGGCATGGTCAGATCCGGACCGCGTTGCGGATCGTGAAAAATAATCGCATCGTTGGGCTTGTCTATCCCGGTCAGCACAGACATATGCCAGCTGCCGGCCGGGGTTTGCCAACCAAACATAATGGGTCCATGGCGACACAAGAGATTGCCGAGTTCATTGGCTGAAAACTGCCTTGATTCCGGTAAACTCACTTCGGCAAGGTTTTCATTTCGCATCAGCCTGAGCACATCTTCACGTTGTTGTAGTCCTTGCGGGCCTGAAGAGTCATAGAGTTCTGGCAGCCCCAGCCGTGGGCCGCTGCTGATGCTATGGCCCAGCATTCTGGTGCAGGCATACCAGCAGCCCATTCGCTCATTCTGTTGAGACACATAAGGTACATGGTTTAAGCGAAAGCCGGCGTTACTGCGAGAGCCGGAACTGCTTCCAGAGTCGTACCCTCTGGACTTTTTACTTTTGCCGAAACATCCGCCAAAAGATGGCGCTGCGGCCGGTAGCTGGGACCAGGGTTTGATGACATCGCCATCGCGGCTTACTTTCTCTGATTGACGAGGCTGGTGCTCGATTACGACAGGGGCGGGGGATGTGAGATGACTGACTTTCAC

**VKVSHLTSPAPVVIEHQPRQSEKVSRDGDVIKPWSQLPAAAPSFGGCFGK** 50
**SKKSRGYDSGSSSGSRSNAGFRLNHVPYVSQQNERMGCWYACTRMLGHSI** 100
**SSGPRLGLPELYDSSGPQGLQQREDVLRLMRNENLAEVSLPESRQFSANE** 150
**LGNLLCRHGPIMFGWQTPAGSWHMSVLTGIDKPNDAIIFHDPQRGPDLTM** 200
**PLDSFNQRLAWRVPHAMLYSEN*?**

>First-NC_013961.1

CTAATTTTCACTGTATAACATGGCGTGTGGCACCCGCCATACCAGACGCTGGTTGAATGAATCCAACGGCATGGTCAGATCCGGACCGCGTTGCGGATCGTGAAAAATAATCGCATCGTTGGGCTTGTCTATCCCGGTCAGCACAGACATATGCCAGCTGCCGGCCGGGGTTTGCCAACCAAACATAATGGGTCCATGGCGACACAAGAGATTGCCGAGTTCATTGGCTGAAAACTGCCTTGATTCCGGTAAACTCACTTCGGCAAGGTTTTCATTTCGCATCAGCCTGAGCACATCTTCACGTTGTTGTAGTCCTTGCGGGCCTGAAGAGTCATAGAGTTCTGGCAGCCCCAGCCGTGGGCCGCTGCTGATGCTATGGCCCAGCATTCTGGTGCAGGCATACCAGCAGCCCATTCGCTCATTCTGTTGAGACACATAAGGTACATGGTTTAAGCGAAAGCCGGCGTTACTGCGAGAGCCGGAACTGCTTCCAGAGTCGTACCCTCTGGACTTTTTACTTTTGCCGAAACATCCGCCAAAAGATGGCGCTGCGGCCGGTAGCTGGGACCAGGGTTTGATGACATCGCCATCGCGGCTTACTTTCTCTGATTGACGAGGCTGGTGCTCGATTACGACAGGGGCGGGGGATGTGAGATGACTGACTTTCAC

**VKVSHLTSPAPVVIEHQPRQSEKVSRDGDVIKPWSQLPAAAPSFGGCFGK** 50
**SKKSRGYDSGSSSGSRSNAGFRLNHVPYVSQQNERMGCWYACTRMLGHSI** 100
**SSGPRLGLPELYDSSGPQGLQQREDVLRLMRNENLAEVSLPESRQFSANE** 150
**LGNLLCRHGPIMFGWQTPAGSWHMSVLTGIDKPNDAIIFHDPQRGPDLTM** 200
**PLDSFNQRLVWRVPHAMLYSEN*?**

>Second-NC_013961.1

CTAATTTTCACTGTATAACATGGCGTGTGGCACCCGCCATGCCAGACGCTGGTTGAATGAATCCAACGGCATGGTCAGATCCGGATCGCGTTGCGGATCGTGAAAAATAATCGCATCGTTGGGCTTGTCTATCCCGGTCAGCACAGACATATGCCAGCTGCCGGCCGGGGTTTGCCAACCAAACATAATGGGTCCATGGCGACACAAGAGATTGCCGAGTTCATTGGCTGAAAACTGCCTTGATTCCGGTAAACTCACTTCGGCAAGGTTTTCATTTCGCATCAGCCTGAGCACATCTTCACGTTGTTGTAGTCCTTGCGGGCCTGAAGAGTCATAGAGTTCTGGCAGCCCCAGCCGTGGGCCGCTGCTGATGCTATGGCCCAGCATTCTGGTGCAGGCATACCAGCAGCCCATTCGCTCATTCTGTTGAGACACATAAGGTACATGGTTTAAGCGAAAGCCGGCGTTACTGCGAGAGCCGGAACTGCTTCCAGAGTCGTACCCTCTGGACTTTTTACTTTTGCCGAAACATCCGCCAAAAGATGGCGCTGCGGCCGGTAGCTGGGACCAGGGTTTGATGACATCGCCATCGCGGCTTACTTTCTCTGATTGACGAGGCTGGTGCTCGATTACGACAGGGGCGGGGGATGTGAGATGACTGACTTTCAC

**VKVSHLTSPAPVVIEHQPRQSEKVSRDGDVIKPWSQLPAAAPSFGGCFGK** 50
**SKKSRGYDSGSSSGSRSNAGFRLNHVPYVSQQNERMGCWYACTRMLGHSI** 100
**SSGPRLGLPELYDSSGPQGLQQREDVLRLMRNENLAEVSLPESRQFSANE** 150
**LGNLLCRHGPIMFGWQTPAGSWHMSVLTGIDKPNDAIIFHDPQRDPDLTM** 200
**PLDSFNQRLAWRVPHAMLYSEN*??**

>Third-NC_013961.1

CTAATTTTCACTGTATAACATGGCGTGTGGCACCCGCCATGCCAGACGCTGGTTGAATGAATCCAACGGCATGGTCAGATCCGGACCGCGTTGCGGATCGTGAAAAATAATCGCATCGTTGGGCTTGTCTATCCCGGTCAGCACAGACATATGCCAGCTGCCGGCCGGGGTTTGCCAACCAAACATAATGGGTCCATGGCGACACAAGAGATTGCCGAGTTCATTGGCTGAAAAATGCCTTGATTCCGGTAAACTCACTTCGGCAAGGTTTTCATTTCGCATCAGCCTGAGCACATCTTCACGTTGTTGTAGTCCTTGCGGGCCTGAAGAGTCATAGAGTTCTGGCAGCCCCAGCCGTGGGCCGCTGCTGATGCTATGGCCCAGCATTCTGGTGCAGGCATACCAGCAGCCCATTCGCTCATTCTGTTGAGACACATAAGGTACATGGTTTAAGCGAAAGCCGGCGTTACTGCGAGAGCCGGAACTGCTTCCAGAGTCGTACCCTCTGGACTTTTTACTTTTGCCGAAACATCCGCCAAAAGATGGCGCTGCGGCCGGTAGCTGGGACCAGGGTTTGATGACATCGCCATCGCGGCTTACTTTCTCTGATTGACGAGGCTGGTGCTCGATTACGACAGGGGCGGGGGATGTGAGATGACTGACTTTCAC

**VKVSHLTSPAPVVIEHQPRQSEKVSRDGDVIKPWSQLPAAAPSFGGCFGK** 50
**SKKSRGYDSGSSSGSRSNAGFRLNHVPYVSQQNERMGCWYACTRMLGHSI** 100
**SSGPRLGLPELYDSSGPQGLQQREDVLRLMRNENLAEVSLPESRHFSANE** 150
**LGNLLCRHGPIMFGWQTPAGSWHMSVLTGIDKPNDAIIFHDPQRGPDLTM** 200
**PLDSFNQRLAWRVPHAMLYSEN*??**

>Fourth-NC_013961.1

CTAATTTTCACTGTATAACATGGCGTGTGGCACCCGCCATGCCAGACGCTGGTTGAATGAATCCAACGGCATGGTCAGATCCGGACCGCGTTGCGGATCGTGAAAAATAATCGCATCGTTGGGCTTGTCTATCCCGGTCAGCACAGACATATGCCAGCTGCCGGCCGGGGTTTGCCAACCAAACATAATGGGTCCATGGCGACACAAGAGATTGCCGAGTTCATTGGCTGAAAACTGCCTTGATTCCGGTAAACTCACTTCGGCAAGGTTTTCATTTCGCATCAGCCTGAGCACATCTTCACGTTGTTGTAGTCCTTGCGGGCCTGAAGAGTCATAGAGTTCTGGCAGCCCCAGCCGTGGGCCGCTGCTGATGCTATGGCCCAGCATTCTGGTGCAGGCATACCAGCAGCCCATTCGCTCATTCTGTTGAGACACATAAGGTACATGGTTTAAGCGAAAGCCGGCGTTACTGCGAGAGCCGGAACTGCTTCCAGAGTCGTACCCTCTGGTCTTTTTACTTTTGCCGAAACATCCGCCAAAAGATGGCGCTGCGGCCGGTAGCTGGGACCAGGGTTTGATGACATCGCCATCGCGGCTTACTTTCTCTGATTGACGAGGCTGGTGCTCGATTACGACAGGGGCGGGGGATGTGAGATGACTGACTTTCAC

**VKVSHLTSPAPVVIEHQPRQSEKVSRDGDVIKPWSQLPAAAPSFGGCFGK** 50
**SKKTRGYDSGSSSGSRSNAGFRLNHVPYVSQQNERMGCWYACTRMLGHSI** 100
**SSGPRLGLPELYDSSGPQGLQQREDVLRLMRNENLAEVSLPESRQFSANE** 150
**LGNLLCRHGPIMFGWQTPAGSWHMSVLTGIDKPNDAIIFHDPQRGPDLTM** 200
**PLDSFNQRLAWRVPHAMLYSEN*??**

>Fifth-NC_013961.1

CTAATTTTCACTGTATAACATGGCGTGTGGCACCCGCCATGCCAGACGCTGGTTGAATGAATCCAACGGCATGGTCAGATCCGGACCGCGTTGCGGATCGTGAAAAATAATCGCATCGTTGGGCTTGTCTATCCCGGTCAGCACAGACATATGCCAGCTGCCGGCCGGGGTTTGCCAACCAAACATAATGGGTCCATGGCGACACAAGAGATTGCCGAGTTCATTGGCTGAAAACTGCCTTGATTCCGGTAAACTCACTTCGGCAAGGTTTTCATTTCGCATCAGCCTGAGCACATCTTCACGTTGTTGTAGTCCTTGCGGGCCTGAAGAGTCATAGAGTTCTGGCAGCCCCAGCCGTGGGCCGCTGCTGATGCTATGGCCCAGCATTCTGGTGCAGGCATACCAGCAGCCCATTCGCTCATTCTGTTGAGACACATAAGGTACATGGTTTAAGCGAAAGCCGGCGTTACTGCGAGAGCCGGAACTGCTTCCAGAGTCGTACCCTCTGGACTTTTTACTTTTGCCGAAACATCCGCCAAAAGATGGCGCTGCGGCCGGTAGCTGGGACCAGGGTTTGATGACATCGCCATCGCGGCTTACTTTCTCTGATTGACGAGGCTGGTGCTCGATTACGACAGGGGCGGGGGATGTGAGACGACTGACTTTCAC

**VKVSRLTSPAPVVIEHQPRQSEKVSRDGDVIKPWSQLPAAAPSFGGCFGK** 50
**SKKSRGYDSGSSSGSRSNAGFRLNHVPYVSQQNERMGCWYACTRMLGHSI** 100
**SSGPRLGLPELYDSSGPQGLQQREDVLRLMRNENLAEVSLPESRQFSANE** 150
**LGNLLCRHGPIMFGWQTPAGSWHMSVLTGIDKPNDAIIFHDPQRGPDLTM** 200
**PLDSFNQRLAWRVPHAMLYSEN*??**

**Multiple Sequence Alignment:** The red highlighted amino acid is the previously studied cys/ser 156 mutation.

5-seq VKVSRLTSPAPVVIEHQPRQSEKVSRDGDVIKPWSQLPAAAPSFGGCFGKSKKSRGYDSG 60

4-seq VKVSHLTSPAPVVIEHQPRQSEKVSRDGDVIKPWSQLPAAAPSFGGCFGKSKKTRGYDSG 60

3-seq VKVSHLTSPAPVVIEHQPRQSEKVSRDGDVIKPWSQLPAAAPSFGGCFGKSKKSRGYDSG 60

2-seq VKVSHLTSPAPVVIEHQPRQSEKVSRDGDVIKPWSQLPAAAPSFGGCFGKSKKSRGYDSG 60

WT VKVSHLTSPAPVVIEHQPRQSEKVSRDGDVIKPWSQLPAAAPSFGGCFGKSKKSRGYDSG 60

1-seq VKVSHLTSPAPVVIEHQPRQSEKVSRDGDVIKPWSQLPAAAPSFGGCFGKSKKSRGYDSG 60

****:************************************************:******

5-seq SSSGSRSNAGFRLNHVPYVSQQNERMGCWYACTRMLGHSISSGPRLGLPELYDSSGPQGL 120

4-seq SSSGSRSNAGFRLNHVPYVSQQNERMGCWYACTRMLGHSISSGPRLGLPELYDSSGPQGL 120

3-seq SSSGSRSNAGFRLNHVPYVSQQNERMGCWYACTRMLGHSISSGPRLGLPELYDSSGPQGL 120

2-seq SSSGSRSNAGFRLNHVPYVSQQNERMGCWYACTRMLGHSISSGPRLGLPELYDSSGPQGL 120

WT SSSGSRSNAGFRLNHVPYVSQQNERMGCWYACTRMLGHSISSGPRLGLPELYDSSGPQGL 120

1-seq SSSGSRSNAGFRLNHVPYVSQQNERMGCWYACTRMLGHSISSGPRLGLPELYDSSGPQGL 120

************************************************************

5-seq QQREDVLRLMRNENLAEVSLPESRQFSANELGNLLCRHGPIMFGWQTPAGSWHMSVLTGI 180

4-seq QQREDVLRLMRNENLAEVSLPESRQFSANELGNLLCRHGPIMFGWQTPAGSWHMSVLTGI 180

3-seq QQREDVLRLMRNENLAEVSLPESRHFSANELGNLLCRHGPIMFGWQTPAGSWHMSVLTGI 180

2-seq QQREDVLRLMRNENLAEVSLPESRQFSANELGNLLCRHGPIMFGWQTPAGSWHMSVLTGI 180

WT QQREDVLRLMRNENLAEVSLPESRQFSANELGNLLCRHGPIMFGWQTPAGSWHMSVLTGI 180

1-seq QQREDVLRLMRNENLAEVSLPESRQFSANELGNLLCRHGPIMFGWQTPAGSWHMSVLTGI 180

************************:***********************************

5-seq DKPNDAIIFHDPQRGPDLTMPLDSFNQRLAWRVPHAMLYSEN*XX 224

4-seq DKPNDAIIFHDPQRGPDLTMPLDSFNQRLAWRVPHAMLYSEN*XX 224

3-seq DKPNDAIIFHDPQRGPDLTMPLDSFNQRLAWRVPHAMLYSEN*XX 224

2-seq DKPNDAIIFHDPQRDPDLTMPLDSFNQRLAWRVPHAMLYSEN*XX 224

WT DKPNDAIIFHDPQRGPDLTMPLDSFNQRLAWRVPHAMLYSEN*X- 223

1-seq DKPNDAIIFHDPQRGPDLTMPLDSFNQRLVWRVPHAMLYSEN*X- 223

**************.**************.**************
