# Supplementary material for: Distinct patterns of natural selection determine sub-population structure in the fire blight pathogen, Erwinia amylovora
Source: Sci Rep. 2019 Sep 30;9:14017. doi: 10.1038/s41598-019-50589-z (PMC6768868; doi:10.1038/s41598-019-50589-z)
Supplement: Supplementary file 8 — Supplementary Figures [file 41598_2019_50589_MOESM8_ESM.docx]

**Supplementary figures corresponding to the manuscript:**

**Distinct patterns of natural selection determine sub-population structure in the fire blight pathogen, *Erwinia amylovora***

Jugpreet Singh^1^, Awais Khan^*1^

^1^Plant Pathology and Plant-Microbe Biology Section, Cornell University, Geneva, NY, 14456, USA

*Corresponding author: Awais Khan, Email: mak427@cornell.edu, Tel: +1 315 787 244

**Table of Content**

Name Page number

Supplementary Figure 1 2

Supplementary Figure 2 3

**
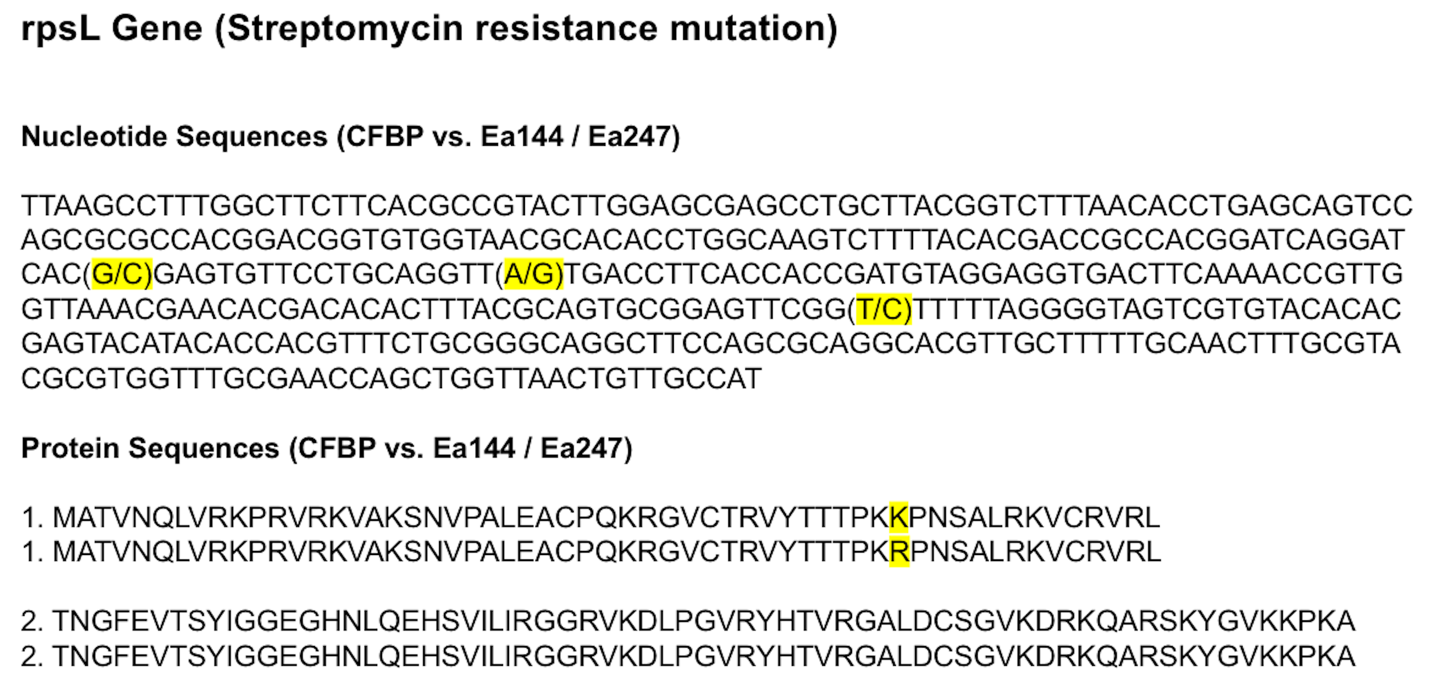
**

**Supplementary Figure 1**. The nucleotide and protein sequences of the rpsL gene associated with streptomycin resistance in *Erwinia amylovora*. The highlighted variants represent the SNPs detected through skim sequencing in this study. A single polymorphism “G/C” causes an amino acid change “K/R” in the protein sequence of rpsL gene.


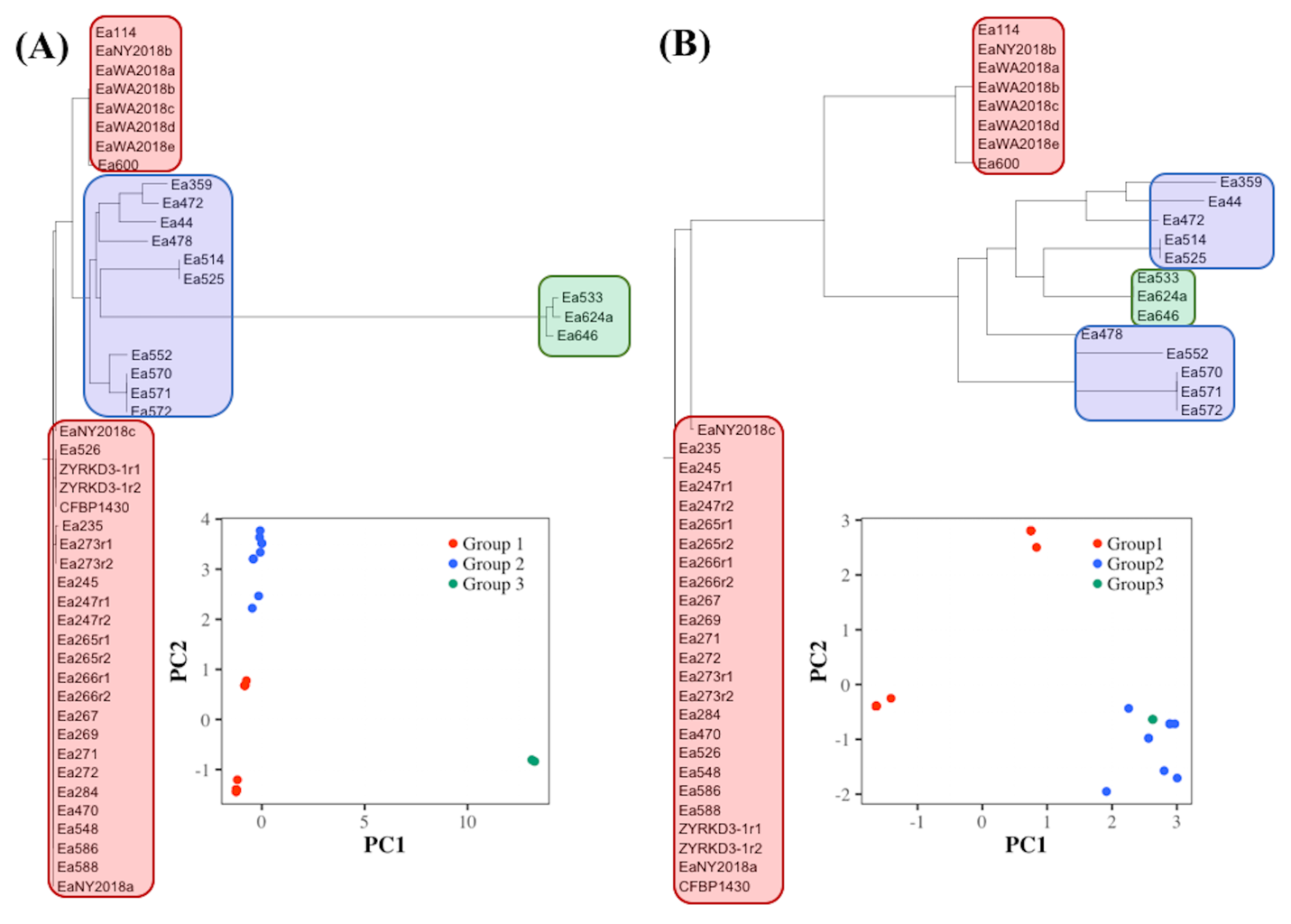


**Supplementary Figure 2.** Principal component and phylogenetic analysis of plasmid variants detected across different *Erwinia amylovora* (EA) strains before (A) and after (B) imposition of minor allele filtering criterion. Different colors represent three different sub-groups in EA strains from population structure analysis of chromosome and plasmid variants. The ‘r1’ and ‘r2’ letters after five strains indicated the two technical replicates for the corresponding strains.
